# Implementation of a Preoperative Nursing Consultation in Elective Paediatric Surgery: An Implementation Science Perspective

**DOI:** 10.1002/nop2.70984

**Published:** 2026-10-30

**Authors:** Juan Manuel Pérez‐Pozuelo, Sonsoles Hernández‐Iglesias, Tania Clemente Gámez, María del Mar Cuart Curtó, Gemma Felis Abadía, Silvia Juaneda Font, Sandra Giao Varela, Sagrario Gómez‐Cantarino

**Affiliations:** ^1^ Faculty of Physiotherapy and Nursing, University of Castilla‐La Mancha Toledo Spain; ^2^ Department of Nursery, Health Sciences Faculty Universidad Francisco de Vitoria, Pozuelo de Alarcón Madrid Spain; ^3^ Villalba General University Hospital Madrid Spain; ^4^ Quirónsalud Palmaplanas Hospital Palma de Mallorca Balearic Islands Spain; ^5^ Rotger Clinic, Quironsalud Group Palma de Mallorca Balearic Islands Spain; ^6^ Quirón Salud University Hospital Madrid Spain

**Keywords:** implementation science, i‐PARIHS, non‐pharmacological techniques, paediatric pain management, paediatric surgery, perioperative nursing, preoperative anxiety

## Abstract

**Aim:**

To analyse the role of standardized protocols and nurse‐led preoperative consultations in elective paediatric surgery and the factors influencing their implementation.

**Design:**

Perspective article guided by the integrated Promoting Action on Research Implementation in Health Services framework.

**Data Sources:**

Structured search of Dialnet, PubMed, SciELO and the Cochrane Library for studies published between 2018 and 2025, conducted between January and March 2025. Of 547 records identified, 40 sources were eligible and 17 inform this article.

**Conclusion:**

Available studies suggest that non‐pharmacological interventions, family involvement and nurse‐led preoperative preparation may reduce anxiety and improve the surgical experience. However, implementation remains inconsistent because of workload, limited training and resources and insufficient institutional support. Nurses are well placed to facilitate the translation of evidence into practice, while organizational culture and leadership determine sustainability.

**Implications for Nursing Practice:**

Nurses can operationalize preoperative consultations through structured, developmentally appropriate assessment of child and parental anxiety and of pain; child‐ and family‐centered education; non‐pharmacological preparation; escalation of high anxiety or pain through agreed referral pathways; and local adaptation of evidence‐based protocols with the multidisciplinary team.

**Impact:**

This article addresses the gap between evidence and practice in paediatric preoperative care. It identifies barriers and facilitators related to the innovation, its recipients, the context and facilitation, and proposes nurse‐led implementation strategies that require evaluation. It is relevant to perioperative nurses, managers and policymakers in paediatric surgical services.

**Reporting Method:**

No EQUATOR reporting guideline applies to perspective articles; the literature search is reported in the Methods.

**Patient or Public Contribution:**

This perspective article is based on a structured literature search and on theoretical implementation frameworks. No primary data were collected, and no patients, caregivers, service users, or members of the public were involved in the design, conduct, analysis, or preparation of this manuscript.

## 1 Introduction

One of the current priorities in healthcare systems is to ensure safe, high‐quality and evidence‐based paediatric surgical care, in line with international recommendations on strengthening the role of nursing. The World Health Organization emphasizes that nurses must be adequately trained, supported and empowered to contribute optimally to the delivery of healthcare services, particularly in complex clinical contexts such as paediatric surgery (World Health Organization 2021). In this field, nurses and paediatric nurse specialists play a fundamental role in the implementation of surgical protocols, health education and the preoperative preparation of children and their families, thereby contributing directly to patient safety and the quality of care (Guamuse‐Pilatasig et al. 2025).

Available studies suggest that the implementation of standardized protocols and nurse‐led paediatric preoperative consultations may be associated with improved clinical outcomes, reduced variability in practice, and lower preoperative anxiety and pain in the paediatric population (Scarano et al. 2021; Villena Rico 2024; Zieliński et al. 2020). In addition, interventions such as the use of non‐pharmacological techniques, parental presence during invasive procedures, and approaches tailored to the child's developmental stage have been reported to benefit the surgical experience and emotional well‐being (Palomares González et al. 2023; Hou et al. 2023). Nevertheless, the literature highlights significant barriers to the systematic adoption of paediatric surgery protocols and the development of structured preoperative consultations, including limited resources, lack of specialized training, insufficient institutional support, and weak clinical leadership. Addressing these challenges is essential to strengthen evidence‐based nursing practice and to ensure safe, high‐quality paediatric surgical care that meets the current and future needs of the paediatric population (Díaz Diñeiro 2022).

## 2 Background

Paediatric surgery constitutes a field of care of particular complexity due to the anatomical, physiological, emotional and cognitive characteristics of the paediatric population. Most surgical procedures in children are performed electively, which allows for careful planning of the intervention and optimization of the preoperative preparation of the child and their family—an element considered key to reducing emotional impact and facilitating understanding of the surgical process (Díaz Diñeiro 2022).

During childhood, surgery is a frequent therapeutic resource for both congenital and acquired conditions, including hernias and digestive or respiratory malformations (Díaz Diñeiro 2022). The perioperative period can represent a highly stressful experience, generating anxiety with psychological and behavioural repercussions that influence the child's cooperation, postoperative recovery and future relationship with the healthcare system (Villena Rico 2024).

Preoperative anxiety is common among paediatric surgical patients and is influenced by factors such as age, cognitive development, temperament, previous medical experiences and parental anxiety (Gutiérrez Gutiérrez 2021; Villena Rico 2024). Higher preoperative anxiety has been associated with greater postoperative pain and postoperative behavioural changes (Villena Rico 2024; Smithers et al. 2024; Alonso Esteban et al. 2024).

The management of anxiety includes both pharmacological and non‐pharmacological interventions. Available studies particularly support the latter—such as preoperative education, distraction techniques and family support—given their reported benefits and favourable safety profile (Scarano et al. 2021; Villena Rico 2024). The combination of both approaches has been reported to improve the overall surgical experience (Scarano et al. 2021).

Parental presence during invasive procedures and anaesthetic induction has been associated with relevant benefits; however, its implementation remains variable and dependent on organizational and attitudinal factors, highlighting the need for clear protocols (Palomares González et al. 2023).

In this context, the humanization of the paediatric surgical process and the role of nursing in preoperative preparation, health education and emotional support for the child–family dyad are fundamental to advancing toward safer, more humanized and evidence‐based paediatric care (Matava et al. 2024; Torres‐Luna et al. 2025).

## 3 Methods

The evidence underpinning this perspective article was identified through a structured literature search on paediatric preoperative nursing consultations, standardized surgical protocols, preoperative anxiety, pain management and patient safety. Controlled descriptors and free‐text terms were combined with Boolean operators and adapted to four databases: Dialnet, PubMed, SciELO and the Cochrane Library. Predefined eligibility criteria (listed below) were applied to ensure the relevance and pertinence of the included sources. The search terms, search strategies and the number of records identified, screened, and included in each database are reported in Table 1.

**TABLE 1:** Structured literature search: Databases, search strategies and selection of sources.

Database	Search terms	Search strategy	Records identified	Records screened (title/abstract)^a^	Eligible sources^b^
Dialnet	Ansiedad; cirugía; niños	Ansiedad AND cirugía AND niños	67	10	5
Dialnet	Ansiedad; niños; prequirúrgico; preoperatorio	Ansiedad AND niños AND (prequirúrgico OR preoperatorio)	42	12	5
Dialnet	Ansiedad; prequirúrgica	Ansiedad AND prequirúrgica	92	7	2
PubMed	Anxiety; surgical; children	Anxiety AND surgical procedure AND paediatric	122	82	15
PubMed	Paediatric; protocol; surgical; pain	Paediatric AND protocol AND surgical AND pain	59	42	5
SciELO	Paediatric; surgical; pain	(Paediatric) AND (surgical) AND (pain)	131	79	4
SciELO	Paediatric; surgical; anxiety	(Paediatric) AND (surgical) AND (anxiety)	23	9	3
Cochrane Library	No farmacológico; anestesia; niños	No farmacológico AND anestesia AND niños	11	1	1
Total			547	242	40
Cited in the article					17

*Note:* Search period: January 2025 to March 2025. Filters applied where available: publication years 2018–2025; Spanish, English or Portuguese; full text available.

^a^ Records retained after (removal of duplicates and of records not meeting the date, language, or full‐text filters); titles and abstracts were screened against the eligibility criteria by (two authors independently), with disagreements resolved by (discussion with a third author).

^b^ Eligible sources after full‐text reading (*n* = 40). Owing to the 25‐reference limit of the Perspective article type, 17 of these were retained and are cited in the article. The remaining eight references cited in the article are theoretical, implementation science and background sources consulted outside the structured search and are not counted in this table.

*Source:* own elaboration.

The search was conducted between January 2025 and March 2025. Database filters for publication date (2018–2025), language, and full‐text availability were applied where available. Of the 547 records identified, 242 were retained for title and abstract screening after removal of duplicates and of records not meeting the filter criteria. Screening against the eligibility criteria was performed by two authors independently, and potentially relevant records were read in full; disagreements were resolved by discussion with a third author. This process yielded 40 eligible sources (Table 1). Given the reference limit of the Perspective article type, the authors then retained the 17 eligible sources most directly relevant to the implementation of paediatric preoperative nursing care, giving priority to the most recent publications and to those published in peer‐reviewed journals; these 17 sources inform the discussion and are cited in the text. No formal quality appraisal was undertaken, consistent with the aim of this perspective article, which is to inform a critical discussion rather than to produce a systematic synthesis of effectiveness. The main findings of the cited sources were extracted, grouped according to recurring themes and organized according to the constructs of the i‐PARIHS framework.

The remaining references (*n* = 8) are implementation science frameworks and literature, books, a doctoral thesis and an institutional document that were purposively consulted to frame the discussion; they did not form part of the structured search and they were not subject to its eligibility criteria.

Inclusion criteria:

- Articles published between 2018 and 2025;
- Publications in Spanish, English and Portuguese;
- Documents with full‐text access;
- Studies that include at least one of the selected descriptors or keywords in the title and abstract;
- Articles focused on paediatric patients;
- Articles addressing anxiety or pain in the context of surgery;

Exclusion criteria:

- Articles whose study population consisted of adults;
- Articles containing non‐relevant data, duplicated information, or information not related to the study topic;

To ground the discussion in implementation theory, this article draws on the integrated Promoting Action on Research Implementation in Health Services (i‐PARIHS) framework (Harvey and Kitson 2016).

Nilsen's taxonomy classifies implementation theories, models and frameworks into process models, determinant frameworks, classic theories, implementation theories and evaluation frameworks (Nilsen 2015). Because the aim of this article is to analyse the factors that enable or hinder the uptake of paediatric preoperative nursing consultations, a determinant framework was considered the most appropriate, in line with recommendations to select frameworks according to the purpose of the work (Moullin et al. 2020). Among the available determinant frameworks, including the updated Consolidated Framework for Implementation Research (CFIR) (Damschroder et al. 2022), i‐PARIHS was selected because it positions facilitation as the active ingredient of implementation—a role that closely mirrors that of nurses in paediatric preoperative care—and because it originated in, and has been widely applied to, nursing practice.

i‐PARIHS proposes that successful implementation results from facilitation activating the innovation with its recipients in their context (Harvey and Kitson 2016). In this framework, the innovation is the evidence‐based practice to be implemented—here, the paediatric preoperative nursing consultation and its components; the recipients are the individuals and teams who adopt, deliver, or are affected by the innovation; and the context comprises the inner setting (the clinical unit and the wider organization) and the outer setting (health‐system policies, regulations and resources).

The discussion is structured around the four i‐PARIHS constructs proposed by Harvey and Kitson (2016), as shown in Figure 1.

**FIGURE 1:**
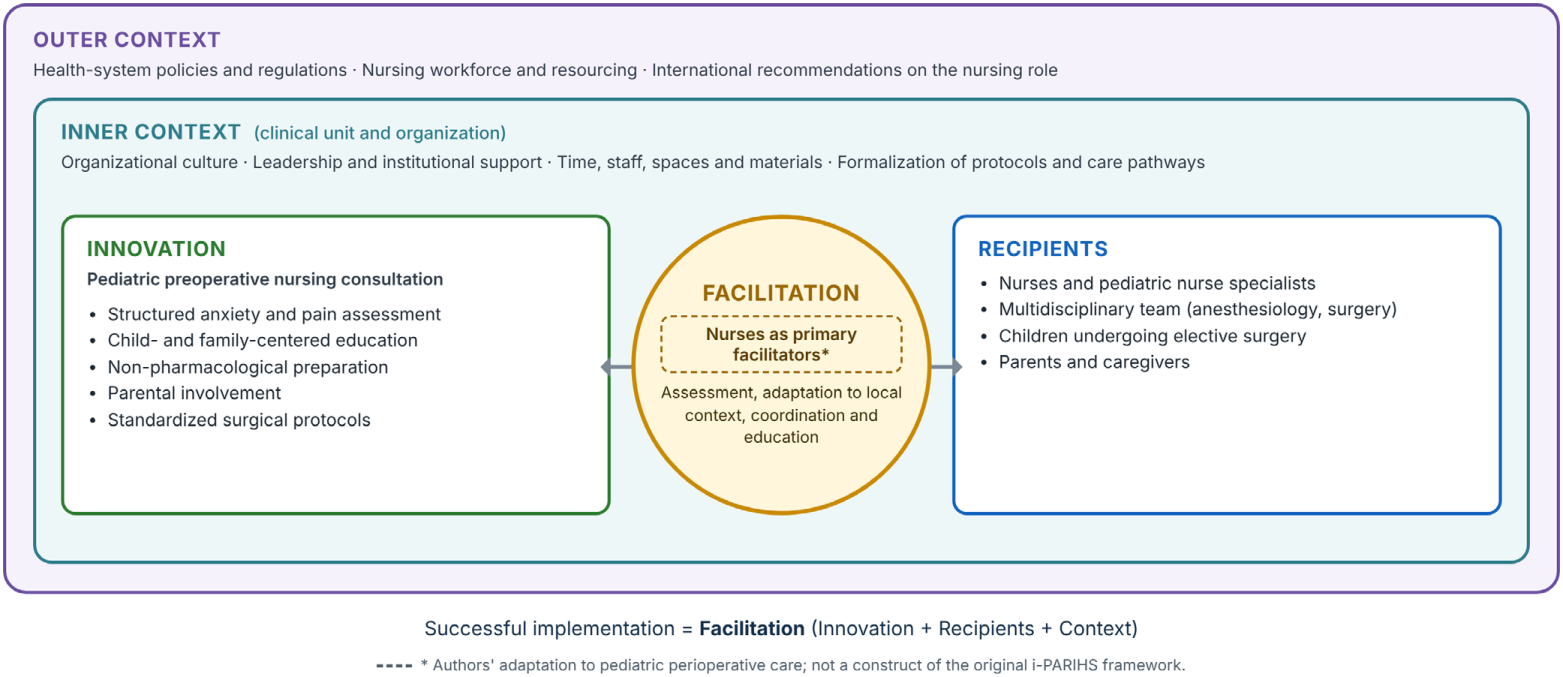
Implementation of a paediatric preoperative nursing consultation interpreted through the i‐PARIHS framework (Harvey and Kitson 2016). The innovation (the paediatric preoperative nursing consultation) and its recipients (nurses, the multidisciplinary team, children and their parents or caregivers) are embedded in the inner context (clinical unit and organization) and the outer context (health‐system policies, workforce and international recommendations). Facilitation acts as the active ingredient linking these constructs. The positioning of nurses as primary facilitators is the authors' adaptation to paediatric perioperative care and is not a construct of the original i‐PARIHS framework.

First, it examines the innovation, that is, the evidence supporting the use of standardized paediatric surgery protocols and non‐pharmacological interventions to reduce preoperative anxiety and improve patient safety (Scarano et al. 2021). Second, it addresses the recipients—particularly nurses and paediatric nurse specialists, as well as children and their families—as key agents in health education, emotional support and family‐centered care (Villena Rico 2024; McCormack and McCance 2017). Third, it considers the inner and outer context, including organizational culture, resource availability and the institutional support required for the implementation of protocols and humanization strategies (Harvey and Kitson 2016). Finally, it examines facilitation as the process that connects these elements.

Facilitation is understood as the driving force that enables the integration of protocols, educational strategies and family participation into routine care through processes of assessment, adaptation and coordination of interventions, thereby promoting evidence‐based, person‐centered clinical practice (Harvey and Kitson 2016). In i‐PARIHS, facilitation can be undertaken by designated facilitators from different professional backgrounds; the positioning of nurses as the primary facilitators of the preoperative consultation is the authors' adaptation of the framework to paediatric perioperative care and is not a construct of the original model.

Although the discussion is relevant to multidisciplinary teams involved in paediatric surgery, the focus of this article is on nursing practice, in line with international recommendations that emphasize the role of nursing in improving quality of care, patient safety and the humanization of healthcare (World Health Organization 2021).

## 4 Discussion

### 4.1 First Construct: Innovation

This construct analyzes the evidence underpinning the innovation, namely interventions aimed at improving paediatric surgical care, with particular attention to reducing preoperative anxiety, pain management, the humanization of care and the integration of non‐pharmacological interventions. From an implementation science perspective, the reviewed literature suggests that, although a growing body of evidence supports these interventions, important challenges remain in their consistent translation into routine clinical practice. In i‐PARIHS, how the innovation is perceived—its underlying evidence, its fit with existing practice and its adaptability to local needs—shapes whether it is adopted (Harvey and Kitson 2016).

The available literature generally supports a comprehensive, person‐centered approach to paediatric surgery, emphasizing the importance of the therapeutic relationship, tailored communication and active family involvement. However, these approaches continue to be implemented heterogeneously and often rely on individual or contextual initiatives, limiting their sustainability and wider adoption (García Nieto et al. 2023). From an i‐PARIHS perspective, this variability reflects the persistent gap between scientific evidence and standardized clinical practice.

The preoperative phase represents a strategic opportunity for implementing evidence‐based interventions, particularly those led by nursing. Health education, emotional preparation and empathic communication have been associated with fewer complications and an improved surgical experience for children and their families. Nevertheless, their routine integration remains limited by workload pressure, lack of time and the absence of structured protocols (Gutiérrez Gutiérrez 2021; Ribera Leclerc et al. 2021), highlighting organizational rather than clinical barriers to implementation.

Preoperative anxiety remains one of the most significant challenges in paediatric surgery because of its high prevalence and clinical consequences. Although validated assessment tools and management strategies are available, they are not consistently incorporated into routine practice, suggesting insufficient systematisation and prioritisation within preoperative care (Scarano et al. 2021; Smithers et al. 2024; López García et al. 2021). This reinforces the need to integrate biopsychosocial assessment more systematically into standardised protocols.

Similarly, paediatric pain management continues to demonstrate a gap between evidence and practice. Despite recommendations supporting multimodal strategies and family involvement, barriers related to professional beliefs, training deficits and insufficient protocolization contribute to the undertreatment of pain (González‐Cárdenas et al. 2018; Zieliński et al. 2020; Ribera Leclerc et al. 2021; Carvalho et al. 2022). These findings underline the importance of implementation strategies targeting both healthcare professionals and organizational structures.

Non‐pharmacological interventions have frequently been reported as effective in reducing anxiety, improving children's cooperation and reinforcing the humanization of care. However, their implementation frequently depends on individual professional motivation rather than standardized organizational processes, limiting scalability and long‐term sustainability (Hou et al. 2023; Lööf and Lönnqvist 2022; Villena Rico 2024; McCormack and McCance 2017). This represents an important opportunity for future research focused on the development of structured implementation strategies.

Overall, this construct suggests that the challenge appears to lie less in the availability of evidence than in its systematic implementation into routine paediatric surgical care. In this context, the i‐PARIHS framework provides a conceptual basis for examining how this translation into standardized and sustainable clinical practice can be facilitated.

### 4.2 Second Construct: Recipients

From an implementation science perspective, the recipients of the intervention constitute a central component of the change process. i‐PARIHS emphasizes that the motivation, values, beliefs, skills and knowledge of those who receive and apply an innovation decisively influence its adoption (Harvey and Kitson 2016).

Nursing professionals occupy a strategic position as both recipients and agents of change in paediatric surgical care. Their continuous contact with the child–family dyad enables them to identify emotional needs, detect anxiety and provide tailored health education. Nurse‐led interventions have been associated with reduced anxiety and an improved surgical experience (Alonso Esteban et al. 2024; Torres‐Luna et al. 2025; Villena Rico 2024). However, their implementation continues to be limited by workload, insufficient specialized training and the lack of formal recognition of the nursing preoperative role.

Within i‐PARIHS, these barriers reflect individual and organizational factors that influence the adoption of evidence‐based interventions. Professionals who perceive clinical usefulness, consistency with their practice and institutional support are more likely to integrate preoperative consultations into routine care (Harvey and Kitson 2016; McCormack and McCance 2017). Conversely, insufficient support and recognition may compromise the long‐term sustainability of these interventions despite the available evidence.

From the perspective of paediatric patients and their families, uncertainty, limited information and insufficient consultation time remain major contributors to preoperative anxiety (Lööf and Lönnqvist 2022). The paediatric preoperative nursing consultation addresses these needs through structured, individualized preparation; however, its availability remains inconsistent, creating inequities in the surgical experience and highlighting the need for more equitable implementation strategies.

Overall, this construct highlights that the successful implementation of paediatric preoperative consultation depends largely on the engagement, competencies and perceptions of its recipients, supporting the need for further research into implementation strategies focused on this level.

### 4.3 Third Construct: Context

The context is a key determinant of the effective implementation of evidence‐based interventions. i‐PARIHS distinguishes the inner context—the local clinical unit and the wider organization—from the outer context, which includes health‐system policies, regulations and resources. The literature emphasizes that organizational culture, available resources and institutional leadership strongly influence the adoption and sustainability of paediatric preoperative consultations (Harvey and Kitson 2016; Braithwaite et al. 2017).

Organizations that promote person‐centered care, patient safety and continuous learning are better positioned to integrate standardized protocols and humanized approaches into paediatric surgery. In contrast, rigid or change‐resistant organizational structures may hinder their implementation despite supportive evidence (McCormack and McCance 2017; Braithwaite et al. 2017).

Resource availability remains another important challenge. Workload, shortages of specialized staff, limited time and inadequate educational spaces or materials may restrict the ability of nursing teams to deliver structured preoperative interventions (World Health Organization 2021). Some interventions, such as virtual parental presence, appear feasible with modest resources and may yield clinical and organizational benefits, although formal economic evaluations are lacking (Matava et al. 2024).

Institutional support is also essential for sustainable implementation. Leadership commitment, protocol formalization and the integration of preoperative consultations into routine care pathways promote standardized practice and interdisciplinary collaboration (Harvey and Kitson 2016). Within i‐PARIHS, adapting interventions to the local context while maintaining fidelity to the evidence is fundamental to overcoming organizational barriers.

Overall, this construct highlights that the successful implementation of paediatric preoperative consultations requires a systemic approach integrating the innovation, its recipients, and the inner and outer context, while supporting future research on sustainable and transferable implementation strategies.

### 4.4 Fourth Construct: Facilitation

In i‐PARIHS, facilitation is the active ingredient that assesses and responds to the characteristics of the innovation, the recipients and the context, combining task‐oriented support with more holistic, enabling approaches (Harvey and Kitson 2016). In paediatric preoperative care, nurses are well placed to undertake this role: they coordinate the preoperative pathway, are in continuous contact with the child–family dyad, and can adapt educational and non‐pharmacological strategies to the child's developmental stage and to local resources (Guamuse‐Pilatasig et al. 2025; Villena Rico 2024).

Positioning nurses as the primary facilitators of the preoperative consultation is the authors' adaptation of i‐PARIHS to this setting rather than a construct of the original framework. Effective nursing facilitation would require protected time, specific training and explicit organizational endorsement of the preoperative nursing role; without these conditions, facilitation is likely to depend on individual motivation, which limits sustainability. The facilitation strategies proposed in this article have not been formally evaluated in paediatric preoperative care and should be tested in future implementation studies.

## 5 Implications for Nursing Practice

The analysis above can be translated into recommendations for perioperative nursing practice. These are presented in two groups, distinguishing practices supported by current evidence from implementation strategies that are proposed on the basis of the i‐PARIHS analysis and require further evaluation.

### 5.1 Practices Supported by Current Evidence

Several components of preoperative nursing care are supported by existing studies and can be incorporated into routine practice: (1) structured assessment of preoperative anxiety and pain in the child, and of anxiety in parents, using validated, age‐appropriate tools (Zieliński et al. 2020; López García et al. 2021); (2) child‐ and family‐centered education adapted to the child's developmental stage and delivered before the day of surgery, including multimedia formats (Hou et al. 2023; Lööf and Lönnqvist 2022); (3) non‐pharmacological preparation, such as therapeutic play, distraction and familiarization with the surgical environment (Scarano et al. 2021; Villena Rico 2024); and (4) facilitation of parental involvement, including parental presence where local policy allows (Palomares González et al. 2023; Matava et al. 2024).

### 5.2 Proposed Implementation Strategies Requiring Further Evaluation

Drawing on the i‐PARIHS analysis, the following strategies are proposed but have not been formally evaluated in this context: (1) designating preoperative nurses as facilitators responsible for coordinating the consultation and adapting evidence‐based protocols to local resources and populations; (2) establishing explicit escalation pathways, agreed with anesthesiology, surgery and, where available, child psychology or play specialists, for children or parents with high anxiety scores or uncontrolled pain concerns; (3) providing protected time and specific training for the preoperative nursing role; and (4) using audit and feedback cycles to monitor uptake, fidelity and child‐ and family‐reported outcomes. The feasibility and effectiveness of these strategies should be tested in future implementation studies.

## 6 Conclusion

The reviewed literature suggests that the implementation of standardized protocols for preoperative management in elective paediatric surgery remains an area under development, with a non‐homogeneous application of the interventions described in the literature. Overall, the analysed studies highlight the relevance of humanized care and the use of non‐pharmacological interventions to reduce preoperative anxiety; however, the diversity of methodological approaches and healthcare contexts, and the absence of a formal quality appraisal limit the ability to draw generalizable conclusions.

Despite these considerations, the literature indicates that preoperative anxiety in children and their families constitutes a clinically relevant problem, with an impact on emotional well‐being, the surgical experience and the safety of the care process. In this regard, preoperative preparation emerges as a key component of more comprehensive care, as it facilitates early assessment, identification of individual needs and the provision of tailored information, as well as the early implementation of non‐pharmacological interventions aimed at reducing perioperative stress.

Nursing plays a fundamental role in this process, both in the development of paediatric preoperative consultations and in health education, emotional support and the personalization of care. Looking ahead, there is a need to advance knowledge generation to better inform the implementation of structured interventions, as well as to develop institutional protocols that integrate paediatric preoperative consultation as a central element of safer, more humanized and child‐ and family‐centered surgical care.

Future research should prioritize the evaluation of implementation strategies and the effectiveness of paediatric preoperative nursing consultations across different healthcare settings, with particular attention to their clinical, organizational and family‐centered outcomes, thereby strengthening the evidence base for their broader implementation and long‐term sustainability.

## Author Contributions

**Juan Manuel Pérez‐Pozuelo:** conceptualization, methodology, project administration, writing – original draft, writing – review and editing, validation, visualization, investigation, supervision. **Sonsoles Hernández‐Iglesias:** methodology, writing – review and editing, writing – original draft. **Tania Clemente Gámez:** methodology, writing – original draft, writing – review and editing. **María del Mar Cuart Curtó:** methodology, writing – original draft, writing – review and editing. **Gemma Felis Abadía:** methodology, writing – original draft, writing – review and editing. **Silvia Juaneda Font:** methodology, writing – original draft, writing – review and editing. **Sandra Giao Varela:** methodology, writing – original draft, writing – review and editing. **Sagrario Gómez‐Cantarino:** methodology, writing – review and editing, writing – original draft.

## Funding

The authors have nothing to report.

## Ethics Statement

The study was conducted in accordance with internationally recognized ethical standards for research integrity and academic scholarship.

## Conflicts of Interest

The authors declare no conflicts of interest.

## Data Availability

Data sharing not applicable to this article as no datasets were generated or analysed during the current study.
